# Is there an association between vitamin D deficiency and adenotonsillar hypertrophy in children with sleep-disordered breathing?

**DOI:** 10.1186/s12887-018-1178-8

**Published:** 2018-06-19

**Authors:** Ji-Hyeon Shin, Byung-Guk Kim, Boo Young Kim, Soo Whan Kim, Sung Won Kim, Hojong Kim

**Affiliations:** Department of Otolaryngology-Head and Neck Surgery, College of Medicine, The Catholic University of Korea, 222 Banpo-daero, Seocho-gu, Seoul, 06591 Republic of Korea

**Keywords:** Vitamin D, Adenoids, Tonsils, Sleep-disordered breathing, Body mass index, Child

## Abstract

**Background:**

Low vitamin D levels have been linked to the risk of sleep-disordered breathing (SDB) in children. Although adenotonsillar hypertrophy (ATH) is the major contributor to childhood SDB, the relationship between ATH and serum vitamin D is uncertain. We therefore investigated the relationship between vitamin D levels and associated factors in children with ATH.

**Methods:**

We reviewed data from all children with SDB symptoms who were treated from December 2013 to February 2014. Of these, 88 children whose serum vitamin D levels were measured were enrolled in the study. We divided the children into four groups based on adenoidal and/or tonsillar hypertrophy. We conducted a retrospective chart review to analyze demographic data, the sizes of tonsils and adenoids, serum 25-hydroxy-vitamin D [25(OH)D] level, body mass index (BMI), and allergen sensitization patterns.

**Results:**

Children in the ATH group had a lower mean 25(OH)D level than did those in the control group (*p* < 0.05). Children with vitamin D deficiencies exhibited markedly higher frequencies of adenoidal and/or tonsillar hypertrophy than did those with sufficient vitamin D (*p* < 0.05). Spearman’s correlation analysis identified an inverse correlation between serum 25(OH)D levels and age, tonsil and adenoid size, and height (all *p* < 0.05). In a multiple regression analysis, tonsil and adenoid size as well as BMI-z score, were associated with 25(OH)D levels after controlling for age, sex, height, and mite sensitization (*p* < 0.05).

**Conclusions:**

Our results suggest that low vitamin D levels are linked to ATH. Both the sizes of the adenoids and tonsils and the BMI-z score were associated with the 25(OH)D level. Therefore, measurement of the serum 25(OH)D level should be considered in children with ATH and SDB symptoms.

## Background

The spectrum of sleep-disordered breathing (SDB) is characterized by snoring, mouth-breathing, and pauses in breathing. SDB includes primary snoring, upper airway resistance syndrome, obstructive sleep apnea (OSA), and obstructive hypoventilation. Children with SDB not only experience sleep disturbances, but also neurocognitive impairment and attention problems. Adenotonsillar hypertrophy (ATH), the primary cause of OSA, is a common childhood disease that can be surgically treated [1–4].

Vitamin D, a fat-soluble vitamin, is synthesized in the skin upon exposure to sunlight and is also obtained from foods. Low vitamin D levels have been linked to many risk factors, including obesity, limited exposure to sunlight, prematurity, malabsorption, darkly pigmented skin, aging, chronic use of steroids or anticonvulsants, and low socioeconomic status [5–7]. In addition, several studies have reported that vitamin D deficiency may increase the risk of numerous acute/chronic otorhinolaryngologic conditions, including allergic rhinitis, chronic rhinosinusitis with nasal polyps, recurrent otitis media, acute respiratory infections, asthma, and benign paroxysmal positional vertigo [8–13].

Chronically low vitamin D levels may also be associated with sleep disorders [14, 15]. Recent studies reported that low vitamin D levels were related to OSA, and that continuous positive airway pressure treatment increased vitamin D levels in adults with OSA [16, 17]. Vitamin D deficiency has been linked to increases in the sizes of the tonsils and/or adenoids and thus to OSA development [18–20]. A decrease in vitamin D levels after an inflammatory insult has also been reported [21, 22], as has an association of low vitamin D levels and adenotonsillar diseases [23, 24]. In contrast, other studies found no association between serum vitamin D levels and such diseases [25, 26]. As the principal cause of vitamin D deficiency is inadequate exposure to sunlight, these conflicting results may be explained by differences in latitude and seasonal variations among studies. In addition, differences in ethnicity and skin color may also be in play [27–29].

In the present study, all subjects lived at the same latitude, were of the same ethnic group, and were evaluated only during winter, therefore reducing potential variations attributable to differences in the abovementioned factors. Our aim was to measure vitamin D levels and analyze associated factors in children with SDB.

## Methods

### Subjects

We conducted a retrospective cross-sectional study at a single, university-based, secondary referral hospital. We recruited all children with SDB symptoms (e.g., snoring, mouth- breathing, paused breathing, and excessive daytime sleepiness) who were treated from December 2013 to February 2014.

In 2012, the authors established critical pathways for the clinical management of SDB, which state that the work up for SDB includes a physical examination, lateral plain X-ray of the nasopharynx, a quality of life evaluation using the Korean version [30] of the obstructive sleep apnea (KOSA)-18 survey [31], allergy evaluation, and measurement of the serum vitamin D level at our outpatient clinic.

The inclusion criteria of the present study were: (1) age 4–12 years; (2) habitual snoring, observed apnea, and/or mouth- breathing during sleep at least 1 year in duration; (3) total KOSA-18 score ≥ 60 (4) evaluation of atopic status using the multiple allergen simultaneous test (MAST); and (5) 25-hydroxy-vitamin D [25(OH)D] level measurement. The exclusion criteria were: (1) any craniofacial anomaly; (2) any anatomical abnormality, including nasal septal deviation, turbinate hypertrophy, and/or nasal polyps; (3) a recent history of nasal or upper airway infection; (4) malnutrition; (5) the use of vitamin D supplements or multivitamin agents; (6) a history of adenoidectomy and/or tonsillectomy; and/or (7) the use of anti-inflammatory and/or anti-allergic drugs within 4 weeks prior to enrollment.

We retrieved demographic, height, body weight, body mass index (BMI), BMI z-score, tonsil and adenoid size, atopic status, and serum vitamin D level data from medical records. We analyzed retrospectively collected data without collecting blood samples by our research group. We described the methods for in vitro IgE sensitization testing and measurement of serum vitamin D levels to clarify how these measurements have been obtained.

BMI was the body weight (kg) divided by the height squared (m^2^). We used the Korean national 2007 growth charts to determine BMI z-scores.

Tonsillar hypertrophy (TH) was graded using the Brodsky scale [32], as follows: grade 0 (tonsils situated in the tonsillar fossa); grade 1 (tonsils just outside of the tonsillar fossa and occupying ≤25% of the airway); grade 2 (tonsils occupying 26–50% of the airway); grade 3 (tonsils occupying 51–75% of airway); and grade 4 (tonsils occupying > 75% of the airway). We used the adenoidal-nasopharyngeal ratio (ANR,) obtained from a lateral plain X-ray of the nasopharynx, to represent the adenoidal size. The depths of the adenoids and nasopharynx were measured using the standard landmarks of Fujioka [33]. The adenoids were measured by drawing lines perpendicular to lines drawn along the straight region of the anterior margin of the basiocciput to the point of maximal adenoidal convexity. The nasopharynx was measured by drawing a line from the anterior inferior edge of the sphenobasioccipital synchondrosis to the posterior superior margin of the hard palate. The ANR was then determined by dividing the first measurement by the second.

We defined grade 3 or 4 tonsils as TH. We defined an ANR ≥ 0.8 as indicative of adenoidal hypertrophy (AH). We then divided the children into four groups: control, AH, TH, and ATH.

### The Korean version of the obstructive sleep apnea (KOSA)-18

To assess quality of life, caregivers completed the KOSA-18 questionnaire, a disease-specific questionnaire validated in Korea. The 18 items of the KOSA-18 are grouped within five domains (sleep disturbance, physical symptoms, emotional distress, daytime function, and caregiver concerns) and are scored using a 7-point ordinal scale, followed by summing of the scores. Possible scores range from 18 to 126 points, with a higher score indicating a worse quality of life. Franco et al. suggested a clinical classification based on the OSA-18, with scores < 60 suggesting a small impact on the health-related quality of life, scores between 60 and 80 a moderate impact, and scores > 80 a large impact [31]. According to this classification, we used the KOSA-18 as one of the inclusion criteria and children with total scores of ≥60 were included in this study.

### Determination of serum 25-hydroxy-vitamin D levels

To evaluate vitamin D status, serum levels of 25-hydroxy-vitamin D (25(OH)D) were measured using a direct competitive chemiluminescence immunoassay (CLIA; LIAISON® 25 OH vitamin D assay; DiaSorin, Saluggia, Italy). The intra- and interassay coefficients of variation for 25(OH)D were 3–6 and 7–11%, respectively.

### Sensitization patterns of the allergens

In vitro IgE sensitization testing was carried out using the multiple allergen simultaneous test (MAST) (RoboScreen™; Bee Robotics Ltd., Gwynedd, UK). The panel consists of 39 allergens, including foods, tree/grass/weed pollens, fungi, dogs, cats, cockroaches, and house dust mites. A score ≥ 2 was interpreted as positive [34].

### Statistical analysis

Statistical analyses were performed using SPSS for Windows software (ver. 15.0; SPSS, Inc., Chicago, IL). Qualitative parameters were evaluated with a chi-square test, and quantitative parameters using a Kruskal-Wallis test. Factors associated with vitamin D deficiency were evaluated using Spearman’s correlation test. For multivariate analysis, a multiple regression analysis was used. All statistical tests were two-tailed. A *P*-value < 0.05 was considered to indicate statistical significance.

### Ethics statement

Written informed consent was not obtained because of the retrospective nature of the study. However, the study protocol was approved by our Institutional Review Board (IRB policy NO. UC15RISI0035).

## Results

**Fig. 1:**
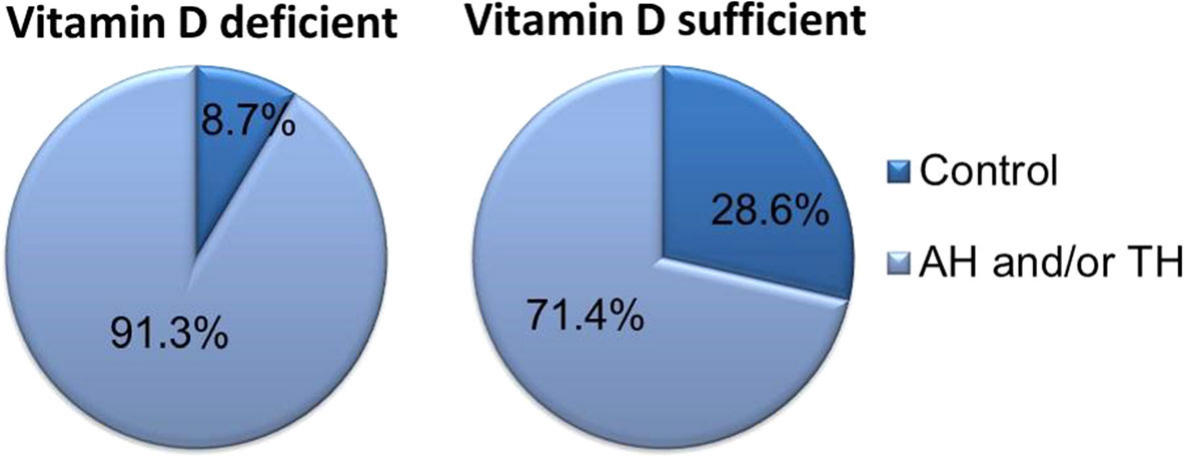
Comparisons of frequencies of adenoid and/or tonsillar hypertrophy by serum 25(OH)D level. Vitamin D-deficient: 25(OH)D < 20 ng/mL; vitamin D-sufficient: 25(OH)D ≥ 20 ng/mL

We included 88 patients [59 males (67.0%) and 29 females (33.0%)] of mean age 8.9 ± 2.5 years. The mean serum 25(OH)D level was 19.4 ± 5.1 ng/mL. A serum 25(OH)D level < 20 ng/mL was considered to reflect a vitamin D deficiency [35]; 52.3% of the children were deficient. The frequency of AH and/or TH in children with vitamin D deficiency and sufficiency was 91.3 and 71.4%, respectively. Deficient children exhibited markedly higher frequency rates of AH and/or TH than did those exhibiting vitamin sufficiency (*p* = 0.035, Fig. 1).

### Children with ATH had lower 25(OH)D levels

**Fig. 2:**
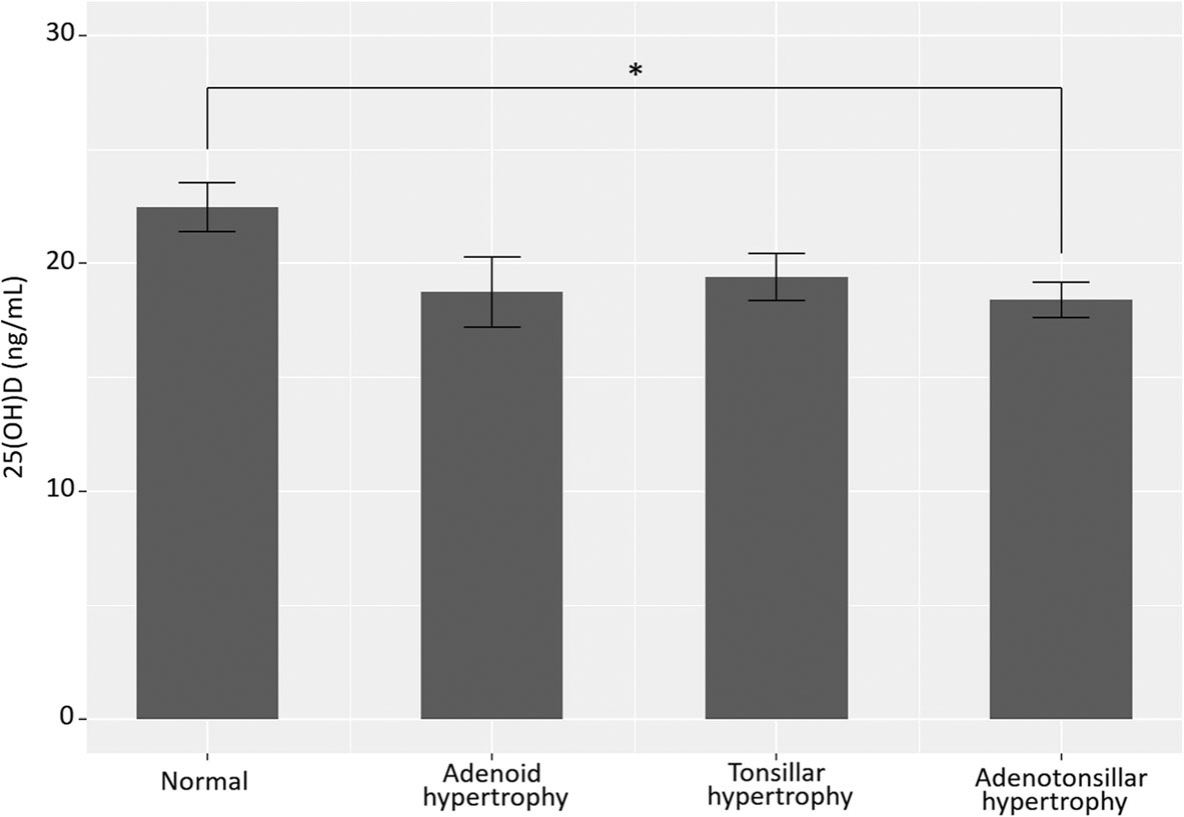
Serum 25(OH)D levels in children with or without adenoid and/or tonsillar hypertrophy. *: *p* < 0.05

We compared the clinical characteristics of the control, AH, TH, and ATH groups. The numbers of children per group were as follows: control, 16 (18.2%); AH, 18 (20.4%); TH, 19 (21.6%), and ATH, 35 (39.8%). The children in the ATH group were younger than those in the AH group (*p* = 0.021). The ATH group had more females than the control and AH groups (*p* = 0.002 and 0.042, respectively). We found no significant difference in height, body weight, BMI, or BMI z-score among the four groups (Table 1). The mean serum 25(OH)D levels of the four groups were as follows: control, 22.5 ± 4.3; AH, 18.7 ± 6.5; TH, 19.4 ± 4.5; and ATH, 18.4 ± 4.5 ng/mL. The children in the ATH group had the lowest mean 25(OH)D level (i.e., lower than that of the control group [*p* = 0.01, Fig. 2]).

**Table 1:** Characteristics of 88 children with or without adenoid and/or tonsillar hypertrophy

	Control (*N* = 16)	Adenoid hypertrophy (*N* = 18)	Tonsillar hypertrophy (*N* = 19)	Adenotonsillar hypertrophy (*N* = 35)
Age (years)	9.0 ± 2.3	10.9 ± 1.5^*^	8.9 ± 2.1	7.8 ± 2.9
Gender (male, %)	14 (87.5%)^*^	14 (77.8%)^*^	13 (68.4%)	18 (51.4%)
Height (cm)	136.7 ± 14.2	141.5 ± 23.9	137.8 ± 14.9	126.5 ± 21.5
Weight (kg)	35.3 ± 13.3	42.2 ± 24.4	40.1 ± 15.9	31.7 ± 18.8
BMI (kg/m^2^)	18.2 ± 3.3	19.6 ± 3.8	20.3 ± 4.1	18.2 ± 4.4
BMI z-score	0.1 ± 1.1	0.5 ± 0.8	0.8 ± 1.1	0.3 ± 1.1
25(OH)D	22.5 ± 4.3	18.7 ± 6.5	19.4 ± 4.5	18.4 ± 4.5

*BMI* body mass index, *25(OH)D* serum 25-hydroxy-vitamin D

^*^versus adenotonsillar hypertrophy group, *p* < 0.05

### Allergen sensitization

A comparison of the atopic status among the four groups showed that the mean number of sensitized allergens in the control, AH, TH, and ATH groups was 3.0, 2.3, 1.5, and 1.2, respectively. The mean was somewhat higher in the control group than in the other groups, but the difference was not significant. The prevalence of atopy in the control, AH, TH, and ATH groups was 50.0, 77.8, 68.4, and 42.9%, respectively. The higher prevalence of atopy in the AH group than in the other groups was also not statistically significant.

### Negative association of age, tonsil size, ANR, and height with serum 25(OH)D

We used Spearman’s correlation test to explore correlations between the serum 25(OH)D level and other variables (Table 2). Age (*r* = − 0.26, *p* = 0.001), tonsil size (*r* = − 0.46, *p* = 0.002), ANR (*r* = − 0.40, *p* = 0.001), and height (*r* = − 0.33, *p* = 0.020) were negatively associated with the serum 25(OH)D level. Body weight, BMI, and BMI z-score also exhibited negative relationships, but these were not statistically significant.

**Table 2:** Correlation coefficients for serum 25(OH)D levels by Spearman’s rank correlation rho

	*r* value	*p* value
Age	− 0.26^*^	0.001
Tonsil size	−0.46^*^	0.002
ANR	−0.40^*^	0.001
Height	−0.33^*^	0.020
Body weight	−0.26	0.060
BMI	− 0.16	0.270
BMI z-score	−0.07	0.580
The number of sensitized allergens	−0.08	0.61

*ANR* adenoidal-nasopharyngeal ratio, *BMI* body mass index

^*^p < 0.05

### Marked association of tonsil size and ANR with serum 25(OH)D

We used a multiple regression analysis to seek factors associated with vitamin D level (Table 3). In model 1, the serum 25(OH)D level was inversely associated with tonsil size (β = − 0.41, *p* = 0.001), ANR (β = − 0.21, *p* = 0.48), and BMI-z score (β = − 1.07, *p* = 0.029) after adjusting for age and sex. These relationships persisted even after further adjustment in model 2 (tonsil size, β = − 0.40, *p* = 0.001; ANR, β = − 0.22, *p* = 0.043; and BMI-z score, β = − 1.07, *p* = 0.001).

**Table 3:** Multiple regression models of serum 25(OH)D level

Parameter	Model 1^a^			Model 2^b^		
	Adjusted OR	95% CI	*p* value	Adjusted OR	95% CI	*p* value
Height	−0.40	−1.35 ~ 0.54	0.408			
Sensitization to mites	−0.13	−0.33 ~ 0.08	0.238			
Tonsil size	−0.41	−0.63 ~ − 0.19	0.001	−0.40	− 0.62 ~ − 0.18	0.001
ANR	− 0.21	−0.43 ~ − 0.01	0.048	−0.22	− 0.43 ~ − 0.11	0.043
BMI-z score	−1.07	−2.38 ~ − 0.23	0.029	− 1.07	− 1.56 ~ − 0.58	0.001

*OR* odds ratio, *CI* confidence interval, *ANR* adenoidal-nasopharyngeal ratio, *BMI* body mass index

^a^Adjusted for age and sex

^b^Adjusted for age, sex, height and sensitization to mites

## Discussion

OSA is associated with an increased risk of vitamin D deficiency. Low vitamin D level increases the risk of OSA by promoting ATH, airway muscle myopathy, and/or chronic rhinitis [23, 36–38]. Recent studies in adults showed that a large proportion of those with OSA also had a vitamin D deficiency [39, 40]. ATH is the most common cause of OSA in children. However, data on the relationship between vitamin D deficiency and AH and/or TH are conflicting [23–26]. In the present study, we used only winter data from children of the same ethnicity (Korean) living at the same latitude (37° 76′ N) to control for contributions made by these factors to the extent of sunlight exposure. We found that the 25(OH)D level was reduced in children with ATH, AH, or TH. The sizes of the adenoids and tonsils, and BMI-z score predicted the serum 25(OH)D level.

We found that 52.3% of all children were vitamin D-deficient. In a nationwide Korean cross-sectional survey, the prevalence of vitamin D deficiency in randomly selected children was 18.4%, thus lower than that in our study. However, the cited survey was conducted in autumn [41]. Another Korean study, conducted in autumn, winter, and spring, found that 59.1% of all children were vitamin D-deficient [42]. These among-study differences are attributable to seasonal variations, participant age, and the prevalence of underlying conditions.

We found that the sizes of the tonsils and adenoids were negatively associated with the serum 25(OH)D level. Several studies have reported relationships between low vitamin D levels and adenotonsillar diseases [23, 24]. A Turkish study found that children with recurrent tonsillitis and allergic rhinitis had significantly lower 1,25-dihydroxyvitamin D [1,25(OH)2D] levels than controls [24]. However, it was not clear that the low vitamin D levels were caused by the tonsillitis or allergic rhinitis, and the seasons in which blood samples were collected were not considered. A pilot study performed in the US found no difference in the vitamin D levels of children undergoing adenotonsillectomies and controls. However, the study included children who underwent adenotonsillectomies not only because of obstruction but also to treat recurrent infections. Again, the seasons in which blood was collected were not reported [25]. As mentioned above, these conflicting results may be explained by differences in latitude, season, ethnicity, and skin pigmentation [6, 29].

Vitamin D deficiency may increase ATH via inadequate regulation of the immune system. Vitamin D receptors are found on T cells, B cells, antigen-presenting cells, macrophages, and dendritic cells. Vitamin D immunomodulates both innate and adaptive immune responses [18, 43]. In terms of the innate immune system, vitamin D increases the production of antimicrobial peptides, including defensin ß and cathelicidin [44, 45]. In the adaptive immune system, the vitamin D inhibits the proliferation of activated lymphocytes, reduces the production of inflammatory cytokines, and promotes the development of induced regulatory T cells [46–48]. Vitamin D deficiency increases the risk of upper and lower airway infections [49, 50]. Many studies have shown that low vitamin D levels are associated with respiratory tract infections and that vitamin D supplements exert beneficial effects during the treatment of infectious diseases [51, 52], although some randomized controlled trials found that vitamin D afforded no benefit in those treated for infectious diseases [53–55]. A recent systematic review and meta-analysis reported that vitamin D supplements had a protective effect against acute respiratory infection, particularly in patients with profound vitamin D deficiency [12]. In terms of the effects of the vitamin D on the adenoids and tonsils, a deficiency may increase recurrent infections. In addition, vitamin D regulates human tonsillar T cells and a deficiency may trigger TH [18, 56]. Interestingly, recent studies suggested that low vitamin D levels are the result rather than the cause of the inflammatory process, as bacterial infection may induce the intracellular conversion of 25(OH)D to 1,25(OH)2D, resulting in high 1,25(OH)2D and low 25(OH)D [57–59]. Therefore, the low vitamin D levels in ATH patients may be a consequence of recurrent adenotonsillitis by bacterial infections.

Many studies have found that increased BMI is associated with vitamin D insufficiency in children [60, 61]. Holick et al. [35] reported that the bioavailability of vitamin D in obesity was reduced because the vitamin was deposited in the body fat. The 2003–2006 USA National Health and Nutrition Examination Survey (which assessed children and adolescents) found that vitamin D deficiency was very prevalent in overweight and obese children [62]. A study of Korean children also revealed that the 25(OH)D level was lower in an overweight compared to a normal-weight group [63]. Consistent with the results of these previous studies, we found that the BMI-z score was negatively associated with the serum 25(OH)D level.

In terms of allergen sensitization, we found no significant difference in either the numbers of allergens to which children were sensitized or the prevalence of atopy among the four groups. Two explanations are possible. One is that the sensitivity of the MAST is low. The other is that children with both allergic rhinitis and turbinate hypertrophy were excluded. Thus, not all children with allergic rhinitis were included. Many studies have found that low vitamin D levels are associated with childhood allergic diseases, including allergic rhinitis, asthma, and atopic dermatitis [64, 65]. A recent Australian study found that a low vitamin D level in early childhood was associated with an increased risk of asthma and early allergic sensitization [65]. In Korea, a recent study showed that low vitamin D levels were associated with symptoms of allergic rhinitis and atopic dermatitis [41]. However, some studies yielded different results [66, 67]. A study of two large birth cohorts found that vitamin D had no protective effect against asthma or allergic rhinitis, and was positively associated with eczema, in 10-year-old children [66]. Thus, no conclusive association has been demonstrated between vitamin D and allergic disease.

A strength of our study is that it was conducted during one season in children of the same ethnicity and living at the same latitude. We thus controlled for several possible confounders. Second, we evaluated allergen sensitization patterns; other similar studies did not [20, 25]. Many studies have reported associations between vitamin D levels and allergic diseases [68, 69]; an evaluation of atopic status is essential when studying the effects of variations in vitamin D levels. Third, we defined clinical features predictive of vitamin D deficiency. Physicians can easily measure the sizes of the tonsils and adenoids, body weight, and BMI in children with SDB.

However, there are some limitations to our study. First, the sample size was too small to allow detailed generalizations to be made. Second, we did not use polysomnography (PSG) for the evaluation of SDB. However, although PSG is the gold standard for the diagnosis of SDB, in practice, the test is time-consuming and cannot be easily performed in all patients. A study in the USA showed that only 10% of children who underwent adenotonsillectomy also underwent a PSG evaluation [70]. In addition, Franco et al. reported that OSA-18 scores correlated significantly with the respiratory distress index determined by PSG [31]. We used the KOSA-18 score [30] as one of the inclusion criteria in our study and included children whose health-related quality of life was moderately to severely affected by OSA. Third, we used the MAST rather than the skin prick test (SPT). However, although the SPT remains a major diagnostic tool, the MAST has the advantage that many allergens can be tested simultaneously. Also, MAST data correlate well with those of the SPT in rhinitis patients, which suggests that the MAST can serve as an alternative to the SPT [71]. Finally, we performed only a retrospective chart review. Additional, larger studies incorporating polysomnographic data may be required before general conclusions can be drawn.

## Conclusions

Approximately half of all children with SDB were vitamin D-deficient. The sizes of the adenoids and tonsils, and BMI-z score were negatively associated with the serum 25(OH)D level. Our results suggest that SDB children with vitamin D deficiencies may need to be evaluated in terms of AH and/or TH, and vice versa.

## Abbreviations

1,25(OH)2D: 1,25-dihydroxyvitamin-D
25(OH)D: Serum 25-hydroxy-vitamin D
AH: Adenoidal hypertrophy
ANR: Adenoidal-nasopharyngeal ratio
ATH: Adenotonsillar hypertrophy
BMI: Body mass index
KOSA-18 survey: Korean version of the obstructive sleep apnea-18 survey
MAST: Multiple allergen simultaneous test
OSA: Obstructive sleep apnea
PSG: Polysomnography
SDB: Sleep-disordered breathing
SPT: Skin prick test
TH: Tonsillar hypertrophy
